# Next-generation sequencing protocol of hematopoietic stem cells (HSCs). Step-by-step overview and troubleshooting guide

**DOI:** 10.1371/journal.pone.0313009

**Published:** 2025-01-09

**Authors:** Justyna Jarczak, Kamila Bujko, Katarzyna Brzeźniakiewicz-Janus, Mariusz Ratajczak, Magdalena Kucia

**Affiliations:** 1 Laboratory of Regenerative Medicine at Medical University of Warsaw, Warsaw, Poland; 2 Department of Hematology, University of Zielona Gora, Multi-Specialist Hospital Gorzow Wlkp., Zielona Gora, Poland; 3 Stem Cell Institute at Graham Brown Cancer Center, University of Louisville, Louisville, KY, United States of America; European Institute of Oncology, ITALY

## Abstract

Populations of very small embryonic-like stem cells (VSELs) (CD34+lin-CD45- and CD133+lin-CD45-), circulating in the peripheral blood of adults in small numbers, have been identified in several human tissues and together with the populations of hematopoietic stem cells (HSCs) (CD34+lin-CD45+) and CD133+lin-CD45+constitute a pool of cells with self-renewal and pluripotent stem cell characteristics. Using advanced cell staining and sorting strategies, we isolated populations of VSELs and HSCs for bulk RNA-Seq analysis to compare the transcriptomic profiles of both cell populations. Libraries were prepared from an extremely small number of cells; however, their good quality was preserved, and they met the criteria for sequencing. We present here a step-by-step NGS protocol for sequencing VSELs and HSC with a description of troubleshooting during library preparation and sequencing.

## 1. Introduction

Among the increasingly frequent transcriptome analyses at the single cell resolution, bulk RNA-Seq is still widely employed, especially because of its relatively low cost and greater simplicity of proceeding. Several examples of RNA-Seq experiments in hematopoiesis studies [1–7] and the developmental journey of hematopoietic stem cells [8–11]. Using RNA-Seq, we can analyze the gene expression level across the entire transcriptome in any experimental setup conditions. Considering the accuracy and affordability of RNA-Seq, it is recognized as the standard technology, especially in molecular biology and genetics [12–14]. The importance of experimental hematology is indisputable, considering the role of hematopoietic stem cells in bone marrow transplantations [3,15–21], immunotherapies [22–24], and in anti-pathogenic and anti-infection processes [24–27]. The dynamics and complexity of the hematopoiesis process involving self-renewal, differentiation, and proliferation require precise, innovative methods such as RNA-Seq to elucidate its molecular basis [7,28–31]. Hematopoietic stem/progenitor cells (HSPCs) are specified into cells from all hematopoietic lineages including erythrocytes, granulocytes, monocytes, lymphocytes, and blood platelets. The major obstacle for RNA Seq in stem cell research is the limited number of primary cells.

The study aimed to evaluate the suitability of HSCs (CD34+lin-CD45+) and VSELs (CD34+lin-CD45-) for use in RNA sequencing and optimize the method of library preparation based on the low number and imperfect quality of RNA samples, isolated from HSC and very small embryonic-like stem cells (VSELs) [32–37]. We wanted to check whether the material with the low number of cells is still sufficient to prepare RNA-seq libraries and to obtain good quality NGS results enabling the analysis of the transcriptomic profile of these cells. In addition, we wanted to analyze the differences in transcriptomic profiles between VSELs and HSC, considering their origin and functions.

## 2. Materials and methods

### 2.1. Samples

Blood samples were obtained from 9 adult COVID patients at the Department of Hematology, Multi-Specialist Hospital Gorzow Wlkp, between 08.01.2023 and 31.03.2023. The information about age, sex, as well as clinical details is presented **in Table 1.** This study was performed based on guidelines and approval of the Medical University of Warsaw Bioethics Committee (permission number KB/50/2022). All procedures were performed in accordance with the Declaration of Helsinki (ethical principles for medical research involving human subjects). All participants gave written consent to participate in the study.

**Table 1:** Characterization of the sample donors including age, sex, and medical diagnosis.

Donor ID	Age	Sex	Infection	Diagnosis
1	67	M	Influenza	Multiple myeloma
2	61	K	COVID	Ovarian cancer
3	69	M	-	-
4	63	K	COVID	-
5	77	M	COVID	Myelodysplastic syndrome
6	39	K	COVID	Hodgkin lymphoma
7	69	M	COVID	Multiple myeloma
8	59	M	COVID	Chronic lymphocytic leukemia
9	76	M	-	Chronic lymphocytic leukemia

### 2.2. Isolation of HSCs and VSELs

Samples were constantly handled at 4 °C, avoiding cell disruption. Blood units, containing between 15–20 mL, were processed with the use of Lysis Buffer (BD) to perform erythrocyte lysis. Samples were incubated at 23°C for 10 minutes and centrifuged for 30 min at 400x g at 4°C. The procedure was repeated two times. The mononuclear phase was collected, washed, and used for further analysis.

### 2.3. Fluorescence-activated cell sorting

The methodology of fluorescence activated cell sorting has been described previously [32,38,39]. MNCs were stained with the following antibodies: Lineage (Lin) cocktail of antibodies, each FITC-conjugated: CD235a (clone GA-R2 [HIR2]), anti-CD2 (clone RPA-2.10), anti-CD3 (clone UCHT1), anti-CD14 (clone M5E2), anti-CD16 (clone 3G8), anti-CD19 (clone HIB19), anti-CD24 (clone ML5), anti-CD56 (clone NCAM16.2) and anti-CD66b (clone G10F5) (all BD Biosciences, San Jose, CA, United States); PE-Cy7-conjugated anti-CD45 (clone HI30, BioLegend, San Diego, CA, United States); and PE-conjugated anti-CD34 (clone 581, BioLegend, San Diego, CA, United States). Antibodies were used in the manufacturer’s recommended concentration. Cells were stained in the dark, placed on ice for 30 min, then washed and resuspended in RPMI-1640 medium containing 2% FBS (Corning Inc, Corning, NY, United States). Cells were sorted according to the following strategy: first, small events (2–15 μm in size) were included in the “lymphocyte like” gate and then further analyzed for the expression of Lin markers and CD45 and CD34 antigens (S1 Fig). Populations of CD34+ VSELs (CD34+lin−CD45−) and HSCs (CD34+lin−CD45+) were sorted on the MoFlo As-trios EQ cell sorter (Beckman Coulter, Brea, CA, United States). 

### 2.4. RNA isolation and quality and quantity assessment

Sorted cells of four cell populations (CD34+lin-CD45- and CD34+lin-CD45+, as well as mononuclear cells (MNC), were used for RNA isolation by RNeasy Micro Kit (Qiagen, Germany). The isolation was supported by the DNA digestion step with the use of the RNase-Free DNase Set (Qiagen, Germany). RNA was eluted in the volume of 15 μL. The isolated RNA samples have been subjected to a qualitative and quantitative assessment using a Quantus Fluorometer (Promega, USA) and TapeStation 4100 (Agilent Technologies, USA), respectively. Universal Human RNA Standard (Agilent Technologies, USA) was used as an internal control of the library preparation process. Information on RNA quantity and quality is presented **in Table 2.**

**Table 2:** Sample characteristics including the number of cells, RNA concentration, and reference mapping results.

L.p.	Sample_ID	Cell type	The number of cells after sorting	RNA conc. (Quantus) (ng/uL)	The concentration of undiluted library (nM)	Number of reads (mln)	Percent of reads aligned to exons (%)
**1**	**1**	**HSC**	2300	1.2	-	-	-
**2**	**2**	**VSEL**	300	1.3	-	84	-
**3**	**3**	**MNC**	-	14.2	24.7	114	63.77
**4**	**4**	**HSC**	7000	1.3	78.9	60	46.64
**5**	**5**	**VSEL**	1800	1.2	-	118	-
**6**	**6**	**MNC**	-	16.2	-	8	-
**7**	**7**	**HSC**	11000	2.1	67.9	30	33.93
**8**	**8**	**VSEL**	1500	2.1	30.5	53	36.67
**9**	**9**	**MNC**	-	2.2	79.5	90	53.74
**10**	**10**	**HSC**	20000	0.79	134.5	67	58.79
**11**	**11**	**VSEL**	2000	0.42	-	33	-
**12**	**12**	**MNC**	-	78.8	1671.5	47	**71.39**
**13**	**13**	**HSC**	10000	0.91	128.2	37	46.51
**14**	**14**	**VSEL**	2000	0.37	-	52	-
**15**	**15**	**MNC**	-	38.7	4980.8	63	45.02
**16**	**16**	**VSEL**	1000	0.17	3.1	0	-
**17**	**17**	**VSEL**	1800	0.58	14.3	30	42.71
**18**	**18**	**VSEL**	350	0.4	-	-	-
**19**	**19**	**VSEL**	2200	lower than blank	-	-	-

### 2.5. Library preparation and sequencing

Illumina Stranded Total RNA Prep Ligation with Ribo-Zero Plus Kit (Illumina, USA) was used to prepare libraries; then they were pooled and run on Illumina NextSeq 1000/2000 (Illumina, USA) in P2 flow cell chemistry (200 cycles) with paired-end sequencing mode assuming 30 millions of reads per sample. Quantity of libraries was measured with the use of the KAPA Library Quantification Kit (Roche, Switzerland), while quality was verified with a High-Sensitivity DNA Kit 1000 on TapeStation 4150 (Agilent Technologies, USA).

### 2.6. Data analysis

The pipeline described by Cebola et al., (https://github.com/CebolaLab/RNA-seq) employed several open-source algorithms for data analysis. Raw BCL files from the Illumina platform were demultiplexed and converted into raw sequences (FASTQ format) with the use of bcl2fastq [40]. Subsequently, the quality of sequences was assessed using fastqc [40], and the reports encompassing sequence quality, GC content, duplication rates, length distribution, K-mer content, and adapter contamination were compiled for all the samples. To remove poor quality sequences and adapter contamination, FASTQ files were trimmed using fastp [41]. Files after trimming were used for alignment to the reference genome (GRCh38.p13), along with a reference transcriptome with the use of a STAR aligner [42]. Files after mapping were sorted, indexed, and flagged with the use of samtools [42] to be ready for final post-alignment quality control with Qualimap [43]. Next, a matrix of gene counts was prepared based on previously aligned BAM files, with the use of Salmon [44]. Count data containing information about the transcripts per million (TPM) values were then imported into R using tximport [45]. The final process of tximport includes combining the transcript-level counts to gene-level for all samples. The use of DEseq2 enabled the analysis of differential gene expression in these data [46]. Data were normalized by the library size and differentially expressed genes (DEGs) were estimated with the use of a Generalized Linear Model (GLM). Data visualization was performed with the use of the ggplot2 R package (version 3.4.4) [47].

First steps of data analysis: pre-processing, quality control, filtering, alignment and reads quantification were done in a Python environment [48]; while differential gene expression and data visualization with the use of R [47], implemented in Anaconda distribution (Anon, 2020. Anaconda Software Distribution, Anaconda Inc. Available at: https://docs.anaconda.com/).

## 3. Results

### 3.1 Quantitative and qualitative assessment of libraries

**Fig 1:**
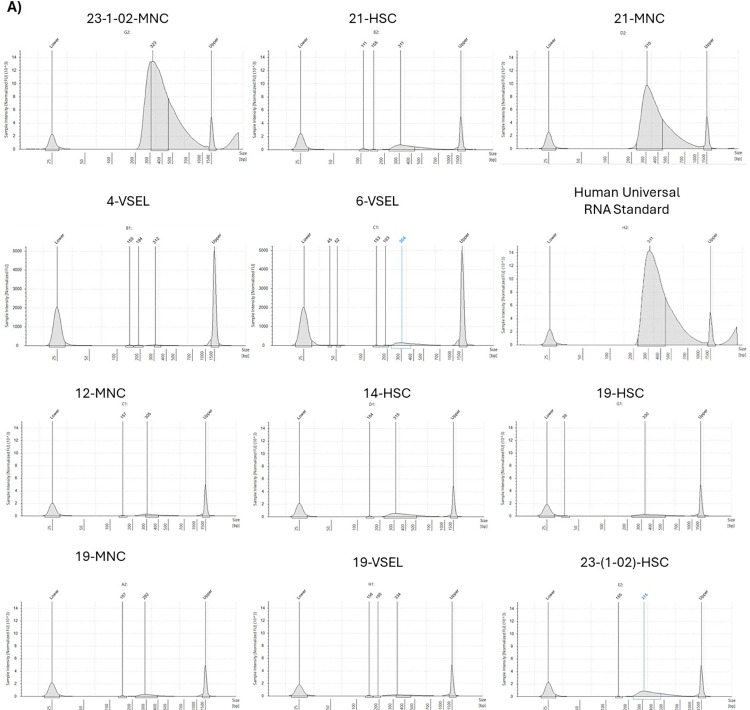
Electropherograms of “good quality” final libraries prepared with the use of Illumina Stranded Total RNA Prep Ligation with Ribo-Zero Plus Kit (Illumina, USA), assessed with the D1000 ScreenTape assay. DNA shows a peak size of around 300 bp, which matches the expected size range between 300 and 400 bp.

**Fig 2:**
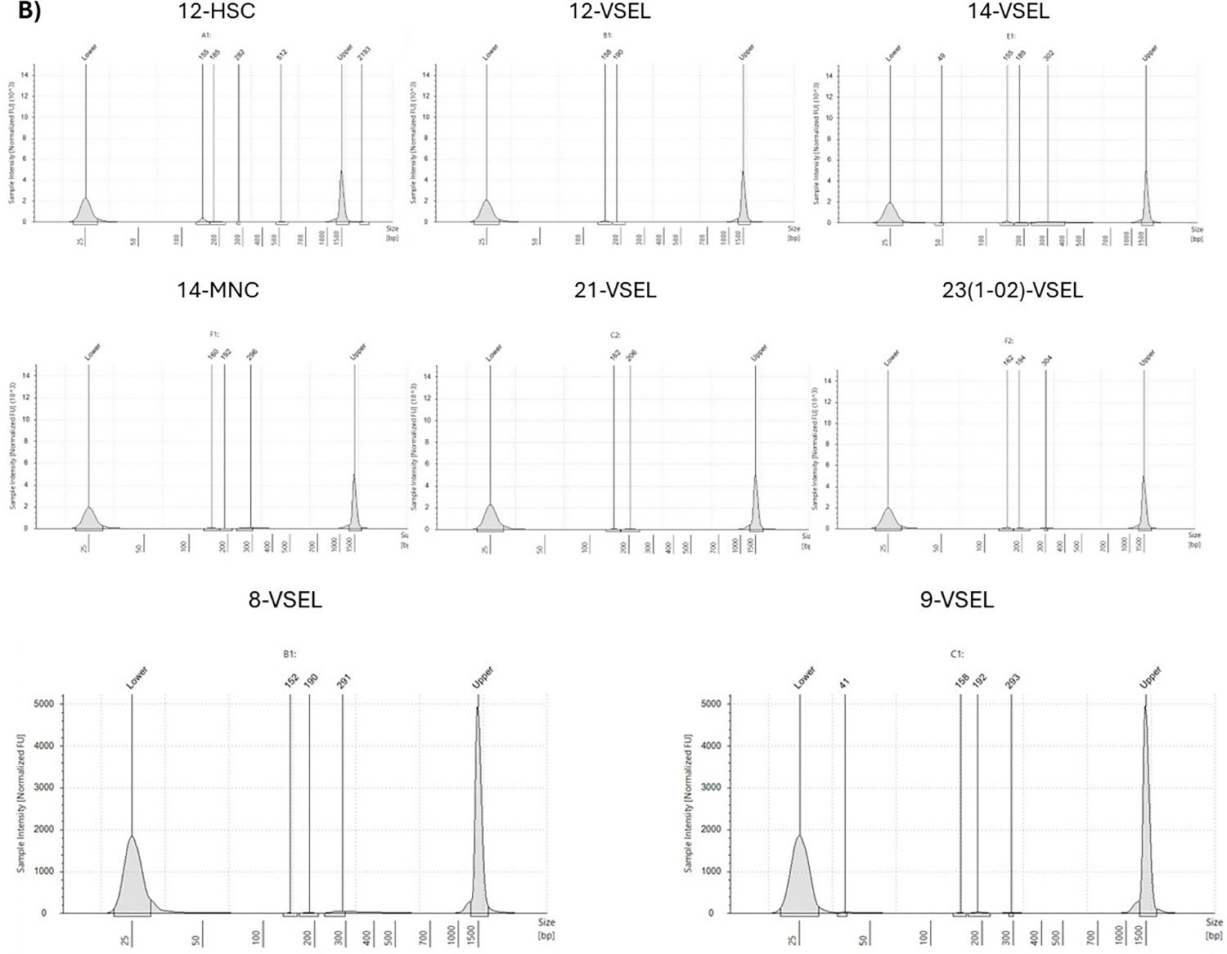
Electropherograms of “bad-quality” final libraries prepared with the use of Illumina Stranded Total RNA Prep Ligation with Ribo-Zero Plus Kit (Illumina, USA), assessed with the D1000 ScreenTape assay. The 300bp peaks, which matches the expected size range are not observed.

First, we wanted to evaluate whether having a low number of cells and a minimal amount of RNA with imperfect quality due to the limited number of sorted cells would allow us to prepare libraries meeting the criteria for next generation sequencing. According to the manufacturer’s instructions, for intact RNA samples, the average fragment length is ~375–475 bp and the expected insert size is ~190 bp. Among 20 libraries, with quality assessed with TapeStation 4150 (Agilent Technologies, USA), 12 (including Human Universal RNA Standard) had a fragment length of more than 300bp and met the standards for sequencing (Fig 1). These were libraries prepared from all three isolated and sorted cell populations: HSCs, VSELs, and MNCs. However, the best results came from libraries prepared from the increased amount of RNA, regardless of the cell type: 23-1-02-MNC, 21-MNC, 14-HSC, and 23-1-02-HSC (Fig 1). On the other hand, we also obtained libraries with bad quality, which were not included in the analysis (Fig 2). Therefore, good quality libraries were next employed for quantitative measurements by KAPA Library Quantification Kit (Roche, Switzerland). The mean library concentration was 1181.69 nM, reaching the maximum for the Human Universal RNA Standard (6966.3 nM), and the lowest value for 4-VSEL sample (3.1 nM). More detailed information on libraries concentration is summarized in **Table 2**.

### 3.2 Quality parameters of sequencing runs and samples after sequencing

**Fig 3:**
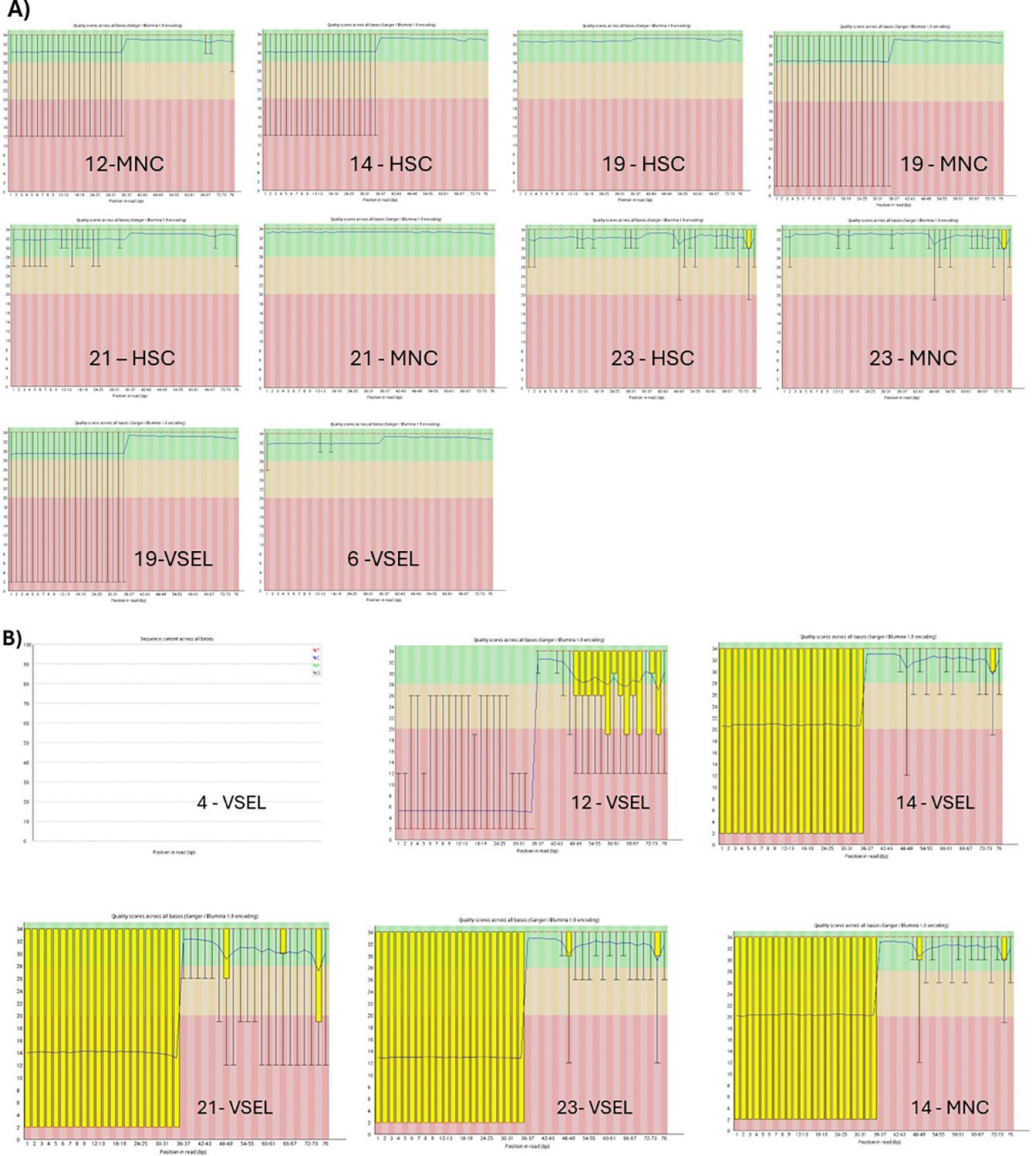
FastQC reports showing per base sequences quality in good (**A**) and bad (**B**) quality samples.

**Fig 4:**
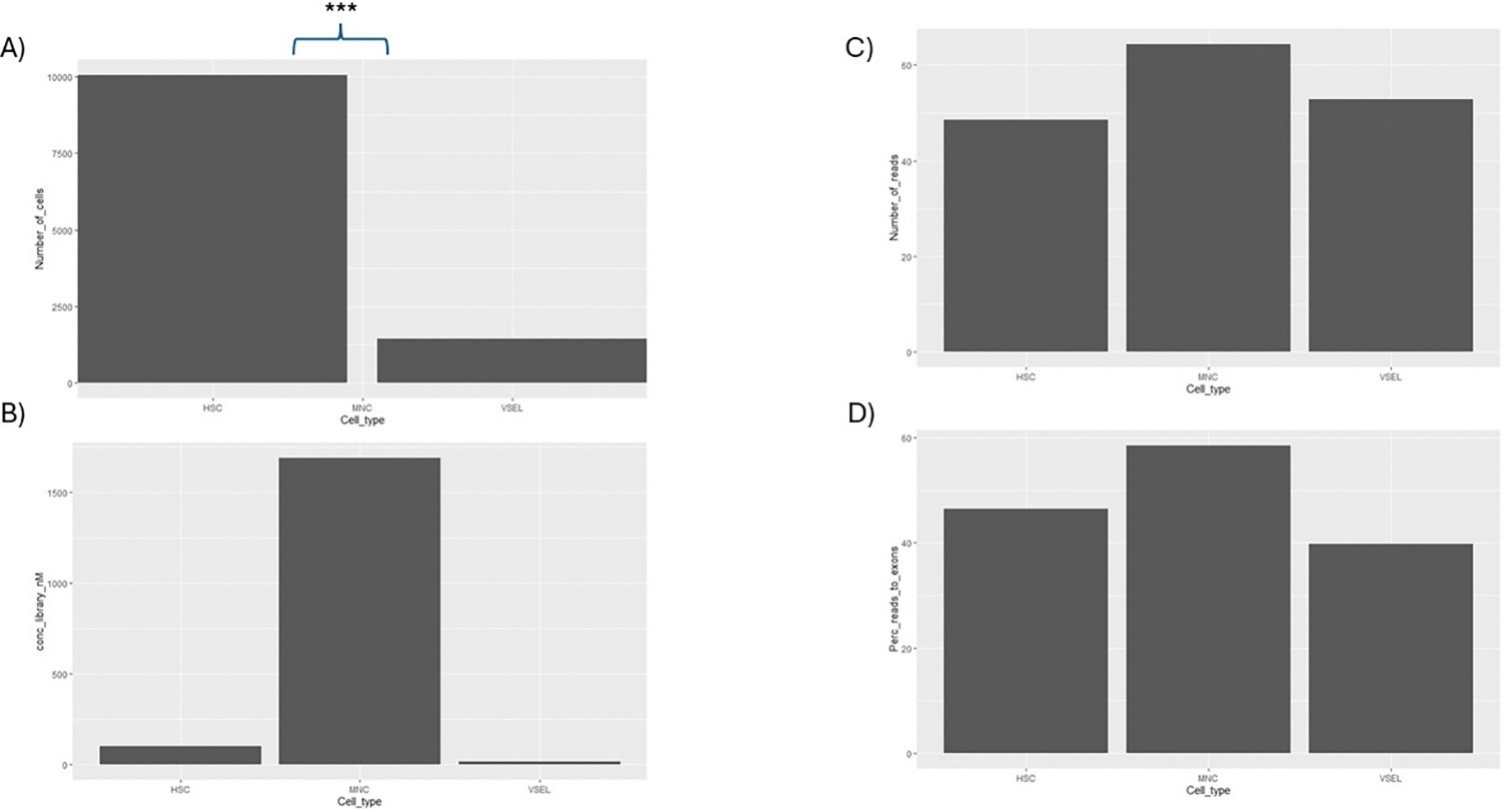
Differences in the cell number (**A**), library concentration (**B**), number of reads (**C**), and percentage of reads aligned to exons (**D**) between HSCs, VSELs, and MNCs.

Libraries were sequenced in two runs (8 samples for 1 run). For the first run, we selected the best samples meeting both qualitative and quantitative criteria: 12-MNC, 14-HSC, 19-HSC, 19-VSEL, 19-MNC, 21-HSC, 21-MNC, and 6-VSEL. The second run included samples with insufficient quality. These were mostly libraries prepared from VSELs: 12-VSEL, 14-VSEL, 21-VSEL, 23(1–02)-VSEL and 4-VSEL as well as MNC: 14-MNC. We also added several other libraries: 23(1–02)-HSC and 23(1–02)-MNC. Both runs had an excellent quality parameter of sequencing with a QC30 parameter greater than 90%. Details are presented in **Table 3**. Despite having good sequencing results, not all samples could be included in the analysis due to their poor-quality parameters (Fig 3B–lower panel, low-quality samples). However, for most samples, we achieved correct sequencing data allowing subsequent analysis (Fig 3A–upper panel, good quality samples). Interestingly, the number of reads for all samples from both runs was close to or greater than the assumed 30 million, except for 4-VSEL (0 reads) and 14-MNC (8 mln of reads) (Table 3). Furthermore, we did not find the statistically significant differences in the number of reads for all cell types (Fig 4).

**Table 3:** Quality parameters of sequencing runs.

Run	Parameter Q30 (%)	Yield (Gbp)	Aligned (%)	Error Rate (%)
**1**	92.93	90.40	5	0.11
**2**	91.73	88.64	4.38	0.27

### 3.3 Mapping parameters

We selected 10 samples for the differential gene expression analysis. Reads were aligned to the human genome reference (GRCh38.p13) and mapping results are presented in Table 2. Approximately, 70–90% of the reads in a high-quality RNA-Seq dataset from a well-annotated genome should align to exons. The mean value evaluated for our samples was equal to 49.9%. The highest values were obtained for 21-MNC (71%) and 12-MNC (63%). This was expected since for those libraries we detected the high RNA concentration and the largest input taken for its preparation (Table 3). Conversely, the lowest values were obtained for 19-HSC (33.93%) and 19-VSEL (36.67). The RNA concentration and input utilized for library preparation were not the lowest. Therefore, the low mapping findings are likely attributable to other factors, such as inadequate RNA quality. However, we did not find any statistical significance in the percentage of reads aligned to exons for all cell types (Fig 4). This high percentage indicates effective mRNA enrichment and minimal contamination. Nonetheless, the precise percentage may fluctuate contingent upon various factors, including the choice of library preparation method, sequencing depth, genome annotation, and biological variability. Of note, it is also crucial to evaluate additional metrics, such as the percentage of reads aligned to genes, introns, and intergenic regions, to comprehensively assess the quality of RNA-Seq data.

### 3.4 Statistical analysis

The number of cells, RNA concentration, library concentration, number of reads, and percentage of reads aligned to exons were compared for HSCs, VSELs, and MNCs. We confirmed the differences in cell number between HSCs and VSELs (MNCs were not included in the comparison), which were isolated from the same blood volume, indicating individual variability (Fig 4, p-value = 0.001). However, the increased number of HSCs did not result in higher RNA concentration (Fig 4). We also did not observe any statistically significant differences in library concentration (except for MNCs), the number of reads, and the percentage of reads aligned to exons between HSCs, VSELs and MNC (Fig 4).

**Fig 5:**
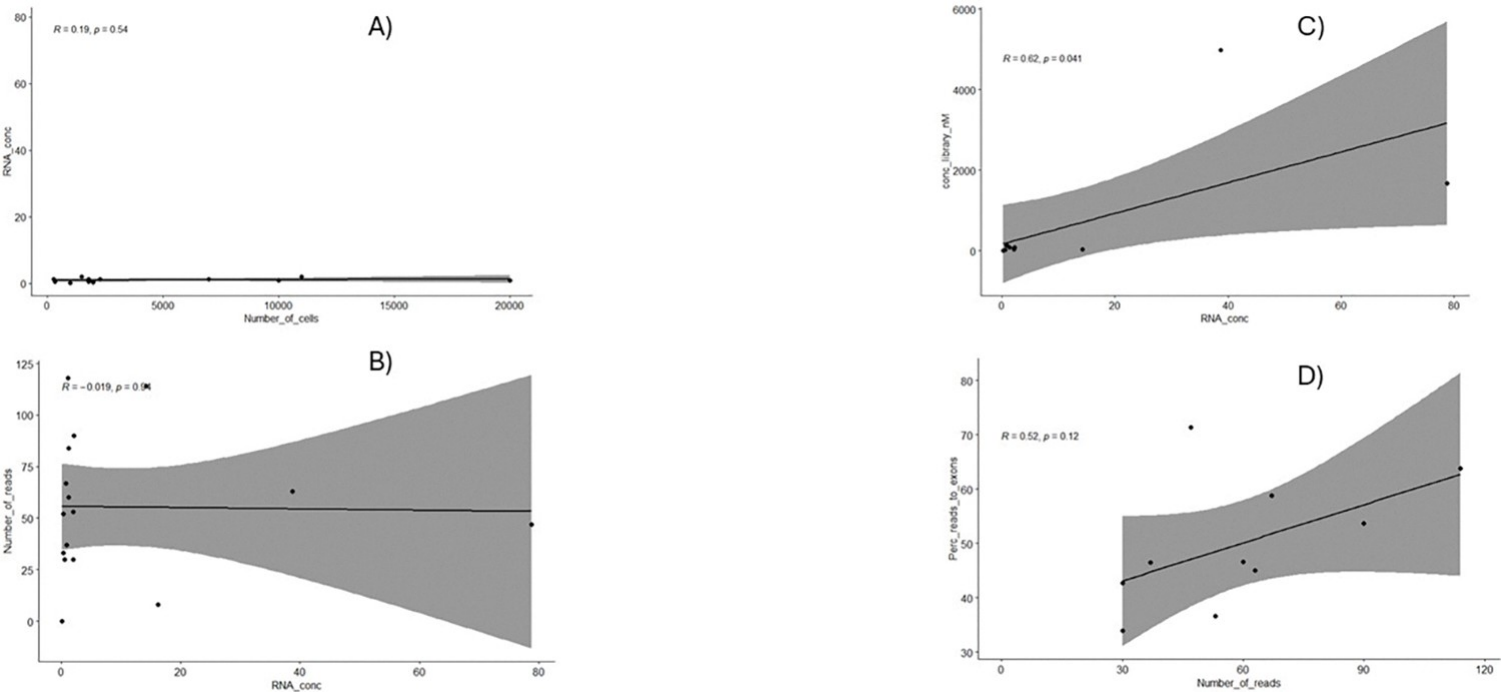
Pearson correlations between RNA concentration and the cell number (**A**), RNA concentration and number of reads (**B**) RNA concentration and library concentration (**C**), and number of reads and percentage of reads aligned to exons (**D**).

In addition, we did not find any statistically significant correlations between all variables (Fig 5) indicating that even samples with low RNA concentration may be considered for analysis and may not achieve low sequencing parameters.

### 3.5 Differential gene expression

Finally, we run differential gene expression analysis using DESeq2, to find the differences in transcriptomic profiles between VSELs and HSCs. MNCs were not included in the analysis. Considering the results of the mapping, it was possible to include 2 VSELs samples and 4 HSCs samples. According to this, we unfortunately observed weak statistical power, the presence of outliers, and results bias. However, this was mostly related to the clinical reasons (patients with CLL) rather than the quality of the prepared libraries.

## 4. Discussion

Using well-established methods with little sequence-specific sample manipulation produces the greatest outcomes in terms of response linearity and reproducibility when sample quantity is not the limiting issue. On the other hand, if gathering such comparatively big samples is not feasible "low-input RNA-seq" is often utilized; however, there are specific challenges and strategies to ensure high-quality data [7,49]. Major challenges are related to the low RNA yield, amplification bias, and dropout events [50].

Although there is some discernible volatility introduced by preamplification processes, the data quality is not significantly affected, and no clear sequence-specific biases are introduced [51,52].

Next, although there are indicators that can provide high levels of HSC purity, HSC populations continue to be functionally diverse in terms of their ability to self-renew after transplantation [53,54]. To methodically describe each cell’s global transcriptional landscape, we employed RNA-seq after multiparameter fluorescence-activated cell sorting (FACS). Principal component analysis was carried out to identify the primary causes of variance (PCA).

The specific research question that an RNAseq experiment is intended to address may or may not be impacted by the fact that bulk tissues may have a mixture of several different cell types. About 0.58 ng of total RNA is the lowest limit of dependable library formation that we have found using the Illumina Stranded Total RNA Prep Ligation with Ribo-Zero Plus kit, and we have used this quantity of RNA in as-yet-unpublished investigations on peripheral blood sorted population of HSC and VSELs.

The quantity of material available for profiling, determines which RNAseq procedures should be used. We discovered that by utilizing the standard Illumina Stranded Total RNA Prep Ligation with Ribo-Zero Plus according to manufacturer procedure, excellent quality libraries may be created with a little less than the producer’s recommended minimum. We found that the Illumina Stranded Total RNA Prep Ligation with Ribo-Zero Plus methodology yields data with a superior linear response to the growing concentration of any given gene when there is enough material available to use it, as opposed to several other "single cell" protocols that perform relatively similarly in this metric.

The techniques and algorithms for RNA-seq are still developing. Many novel applications of RNAseq-based analysis are now feasible as a result of advancements. There are differences in the essential statistical methodologies and concerns for sample-level, gene-level, transcript-level, and exon-level applications. Given that various statistical models and data distribution assumptions are frequently employed by tools to address identical biological questions. The agreement between the results varies so frequently. The final interpretation may differ depending on the parameters and tool selection. The results can potentially be affected by bias resulting from the sequencing library, such as bias resulting from short reads or insufficient small non-coding RNA detection bias caused by RNA modification [42].

To sum up, we outlined the developing process for analysis based on bulk RNAseq and emphasized the information gaps. Obtaining agreement on the most appropriate instruments or pipelines is difficult because biological goals and situations vary. We offer a useful guide of key limitations and quality control analysis that should be addressed when working with low input RNA Seq.

## 5. Conclusions

The article discusses techniques and strategies for optimizing RNA sequencing in scenarios with limited cell numbers and compromised sample quality. It highlights the importance of careful sample handling and robust data analysis approaches to mitigate the challenges posed by low-input and degraded RNA samples. Importantly, the study emphasizes that even working with stem cells and their low number do not have to disqualify samples from RNA-Seq analysis.

## Supporting information

S1 Fig Cell sorting strategy for the isolation of HSCs and VSELs from peripheral whole blood. Immunostained cells were first visualized by dot plot showing forward scatter (FSC) vs. side scatter (SSC) signals, where small events ranging from 2–15 μm were gated (P1) (**A**) and further analyzed for the expression of Lineage markers. Lineage negative events were gated (Lin-) (**B**) and analyzed for the expression of CD45 and CD34 antigens. The populations of CD34+Lin-CD45+ HSCs (**C**) and CD34+Lin-CD45- VSELs (**D**) were separately sorted on the MoFlo Astrios EQ cell sorter. MNCs isolation and staining was described in the Materials and Methods section. Representative dot plots saved during the sample acquisition are shown.
(TIF)
