# Supplementary figures and images for: Next-generation sequencing protocol of hematopoietic stem cells (HSCs). Step-by-step overview and troubleshooting guide
Source: PLoS One. 2025 Jan 9;20(1):e0313009. doi: 10.1371/journal.pone.0313009 (PMC11717189; doi:10.1371/journal.pone.0313009)

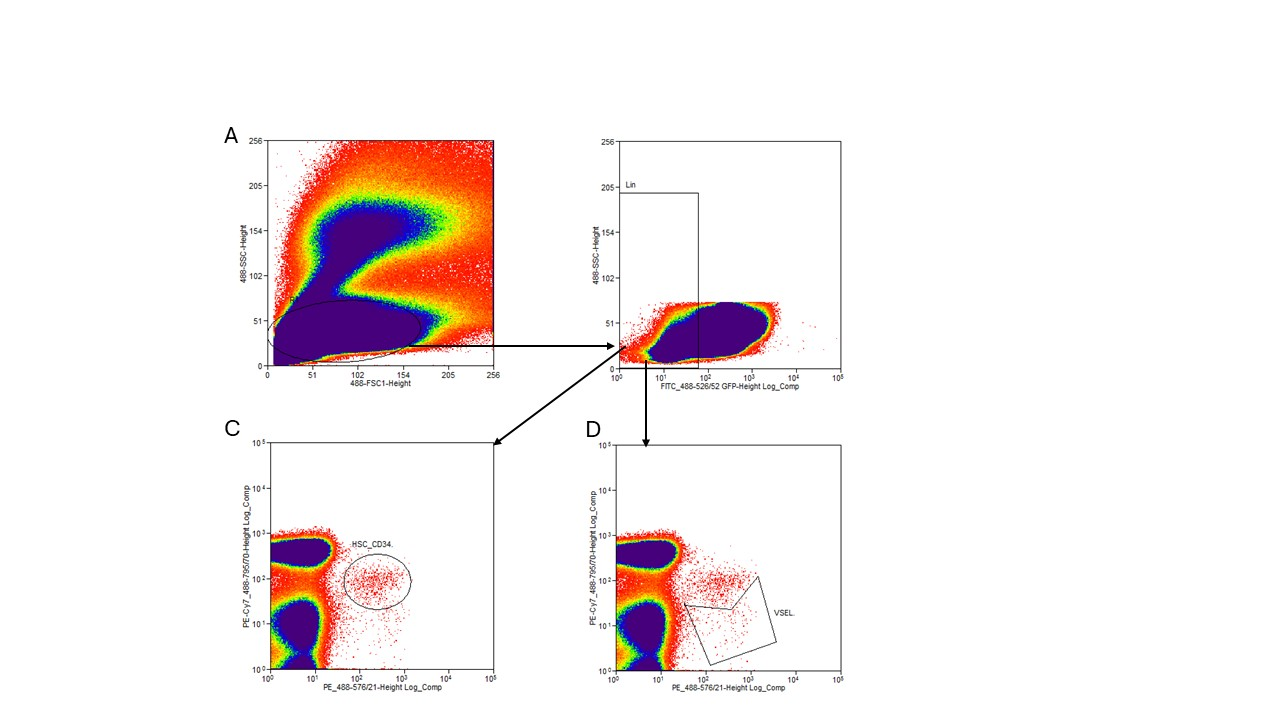

Supplement: S1 Fig — Immunostained cells were first visualized by dot plot showing forward scatter (FSC) vs. side scatter (SSC) signals, where small events ranging from 2–15 μm were gated (P1) (A) and further analyzed for the expression of Lineage markers. Lineage negative events were gated (Lin-) (B) and analyzed for the expression of CD45 and CD34 antigens. The populations of CD34+Lin-CD45+ HSCs (C) and CD34+Lin-CD45- VSELs (D) were separately sorted on the MoFlo Astrios EQ cell sorter. MNCs isolation and staining was described in the Materials and Methods section. Representative dot plots saved during the sample acquisition are shown. (TIF) [file pone.0313009.s001.tif]
